# Correction: Somoza et al. Microfluidic Fabrication of Gadolinium-Doped Hydroxyapatite for Theragnostic Applications. *Nanomaterials* 2023, *13*, 501

**DOI:** 10.3390/nano16070391

**Published:** 2026-03-24

**Authors:** Manuel Somoza, Ramón Rial, Zhen Liu, Iago F. Llovo, Rui L. Reis, Jesús Mosqueira, Juan M. Ruso

**Affiliations:** 1 Soft Matter and Molecular Biophysics Group, Department of Applied Physics, University of Santiago de Compostela, 15782 Santiago de Compostela, Spain; 2 3B’s Research Group, I3Bs—Research Institute on Biomaterials, Biodegradables and Biomimetics, University of Minho, Headquarters of the European Institute of Excellence on Tissue Engineering and Regenerative Medicine AvePark—Parque de Ciência e Tecnologia Zona Industrial da Gandra Barco, 4805-017 Guimarães, Portugal; 3 ICVS/3B’s—PT Government Associate Laboratory, 4806-909 Braga, Portugal; 4 Department of Physics and Engineering, Frostburg State University, Frostburg, MD 21532, USA; 5 QMatterPhotonics, Departamento de Física de Partículas, Universidade de Santiago de Compostela, 15782 Santiago de Compostela, Spain; 6 Institute of Materials (iMATUS), Department of Applied Physics, Universidade de Santiago de Compostela, 15706 Santiago de Compostela, Spain

**Error in Figure**

Upon careful review, we realized that a mistake was made in the initial submission [1], where we unintentionally included the same image for both Figure 3a and Figure 3c. We want to clarify that these are actually two different images, and the image previously labeled as Figure 3c was incorrectly repeated. After reviewing the data from our analysis, including the image treatments and the calculations of fractal dimension and roughness, we can confirm that the corrected image now provided is the accurate version for Figure 3c. The updated version of Figure 3, with the proper image, is now included. The authors state that the scientific conclusions are unaffected. This correction was approved by the Academic Editor. The original publication has also been updated.

## Figures and Tables

**Figure 3:**
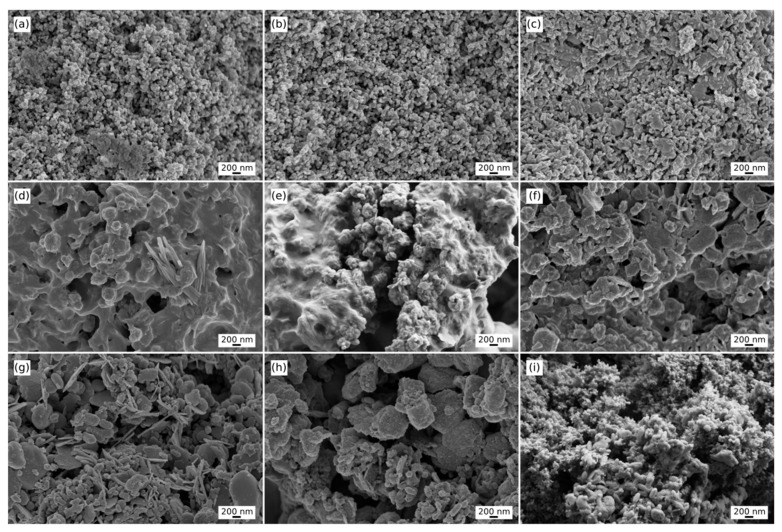
SEM images of the different samples. Images from the upper row (**a**–**c**) depict the surface of pure HAp nanorods. The central row (**d**–**f**) corresponds to the HAp:GD1 samples, and the lower row (**g**–**i**) represents the results for the HAp:GD10 material. It can be seen in the untreated images that the incorporation of gadolinium causes softness in the appearance of protuberances and a general change in the overall surface.
