# Supplementary material for: Microfluidic Fabrication of Gadolinium-Doped Hydroxyapatite for Theragnostic Applications
Source: Nanomaterials (Basel). 2023 Jan 26;13(3):501. doi: 10.3390/nano13030501 (PMC9921701; doi:10.3390/nano13030501)
Supplement: Supplementary file 1 [file nanomaterials-13-00501-s001.zip › nanomaterials-2183647-supplementary.pdf]

Supplementary material

Microfluidic Fabrication of Gadolinium-Doped Hydroxyapatite for Theragnostic Applications

Manuel Somoza [1], Ramón Rial [1,2,3,*], Zhen Liu [4], Iago F. Llovo [5,6], Rui L. Reis [2,3], Jesús Mosqueira [5,6] and Juan M. Ruso [1]

[1] Soft Matter and Molecular Biophysics Group, Department of Applied Physics, University of Santiago de Compostela, 15782 Santiago de Compostela, Spain
[2] 3B's Research Group, I3Bs—Research Institute on Biomaterials, Biodegradables and Biomimetics, University of Minho, Headquarters of the European Institute of Excellence on Tissue Engineering and Regenerative Medicine AvePark—Parque de Ciência e Tecnologia Zona Industrial da Gandra Barco, 4805-017 Guimarães, Portugal
[3] ICVS/3B's—PT Government Associate Laboratory, 4806-909 Braga, Portugal
[4] Department of Physics and Engineering, Frostburg State University, Frostburg, MD 21532, USA
[5] QMatterPhotonics, Departamento de Física de Partículas, Universidade de Santiago de Compostela, 15782 Santiago de Compostela, Spain
[6] Institute of Materials (iMATUS), Department of Applied Physics, Universidade de Santiago de Compostela, 15706 Santiago de Compostela, Spain
* Correspondence: ramon.rial@usc.es

Tables

Table S1. Employed quantities for the different stocks A_i. Concentrations were adjusted to meet the proposed ratios between Ca and Gd.

	m_{CaCl_2} [g]	m_{GdCl_3} [g]
Ca	0.1714	-
Ca:Gd (1:1)	0.0857	0.2871
Ca:Gd (1:10)	0.0039	0.1305

Table S2. Employed quantities for the stock B and C.

	V [mL]	m [g]
PPG	1.56	-
CTAB	-	0.031
$NaNO_2$	-	2.151
Na_3PO_4	-	0.413

Table S3. Quantities and concentrations of each compound during the microfluidic synthesis process. The "i" subscript denotes the values corrresponding to the outflow of the first microchip, "m" represent the values at the entrance of the second microrreactor and "f" corresponds to the final mixture.

	M [g mol^{-1}]	V [mL]	m [g]	n [mol]	ρ [g mL^{-1}]	V_i [mL]	$[M]_i$ [mol L^{-1}]	V_m [mL]	$[M]_m$ [mol L^{-1}]	V_f [mL]	$[M]_f$ [mol L^{-1}]
PPG	425	0.39	-	$92 \cdot 10^{-5}$	1.004	5	$184.1 \cdot 10^{-3}$	10	$92 \cdot 10^{-3}$	15	$61.4 \cdot 10^{-3}$
CTAB	364.45	-	$7.8 \cdot 10^{-3}$	$2.1 \cdot 10^{-5}$	-	5	$4.3 \cdot 10^{-3}$	10	$2.1 \cdot 10^{-3}$	15	$1.4 \cdot 10^{-3}$
$NaNO_2$	69	-	$537.6 \cdot 10^{-3}$	$779.2 \cdot 10^{-5}$	-	5	$1558.4 \cdot 10^{-3}$	10	$779.2 \cdot 10^{-3}$	15	$519.5 \cdot 10^{-3}$
$CaCl_2$	110.98	-	$42.9 \cdot 10^{-3}$	$38.6 \cdot 10^{-5}$	-	5	$772.3 \cdot 10^{-3}$	10	$38.6 \cdot 10^{-3}$	15	$25.7 \cdot 10^{-3}$
Na_3PO_4	163.94	-	$103.3 \cdot 10^{-3}$	$63 \cdot 10^{-5}$	-	-	-	5	$124 \cdot 10^{-3}$	15	$41.9 \cdot 10^{-3}$

Figures

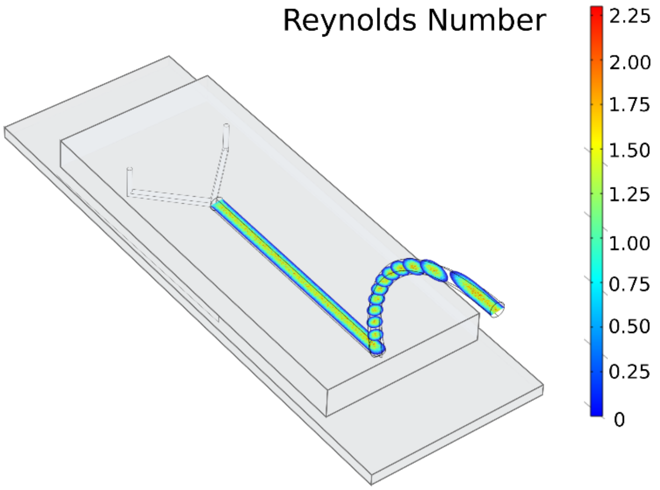

Figure S1. Reynolds number values inside the channel of the first microreactor. The low Reynold values probe that laminar flow is assured all through the process.

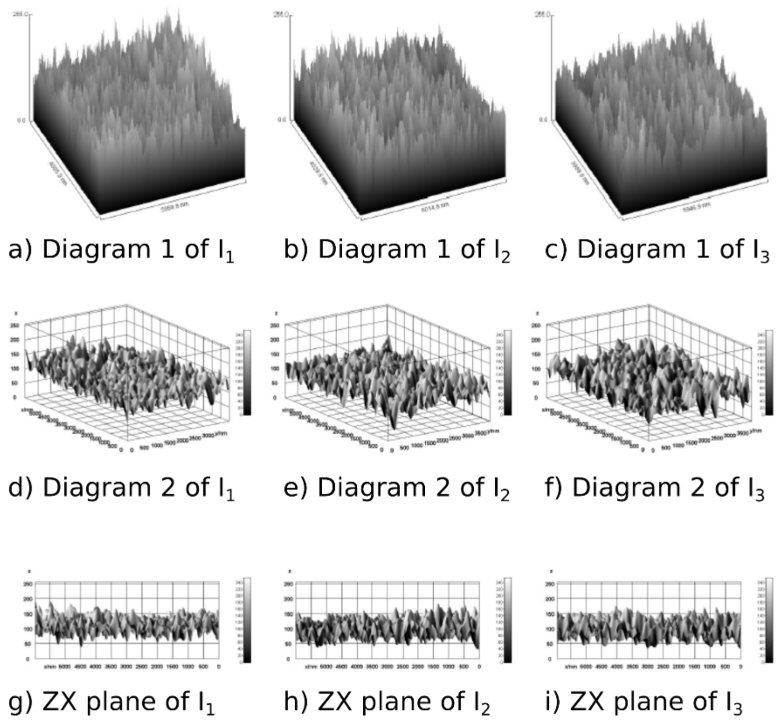

Figure S2. FE-SEM Image processing for pure HAp.

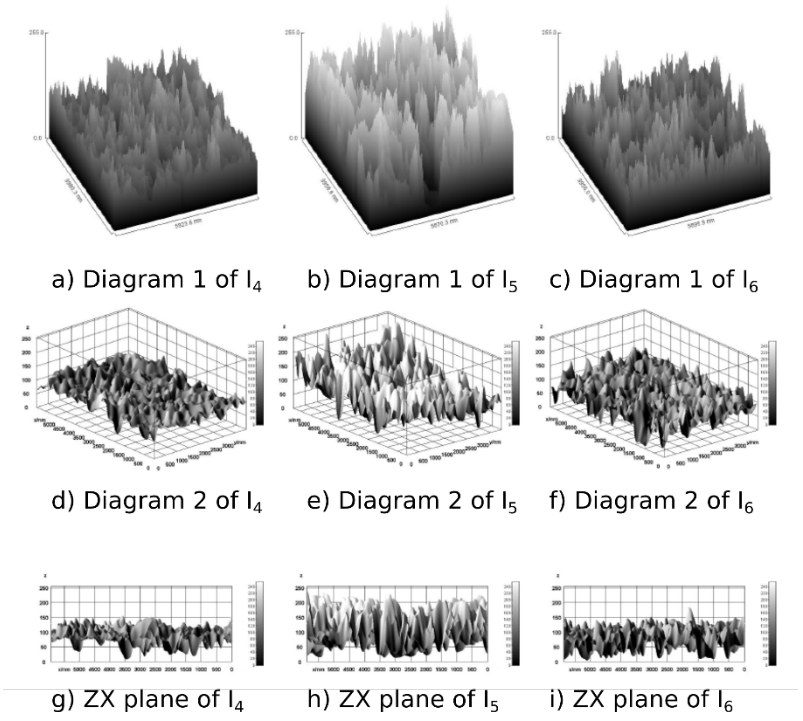

Figure S3. FE-SEM Image processing for HAp:Gd1.

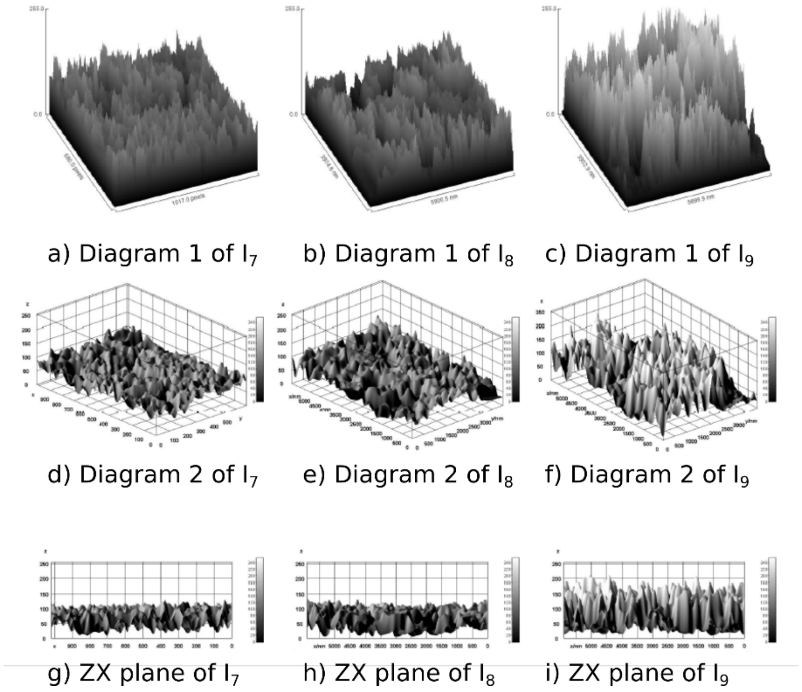

Figure S4. FE-SEM Image processing for HAp:Gd10.

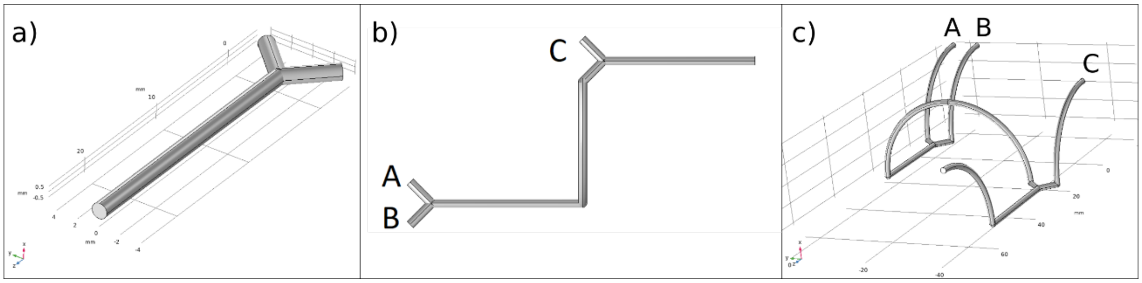

Figure S5. Chip disposition. Figure a) shows the tridimensional representation of the simulated microchip. Image b) represents the two microrreactor conected in series and Figure c) shows the 3D model used for the Computational Fluid Dynamics of the system.

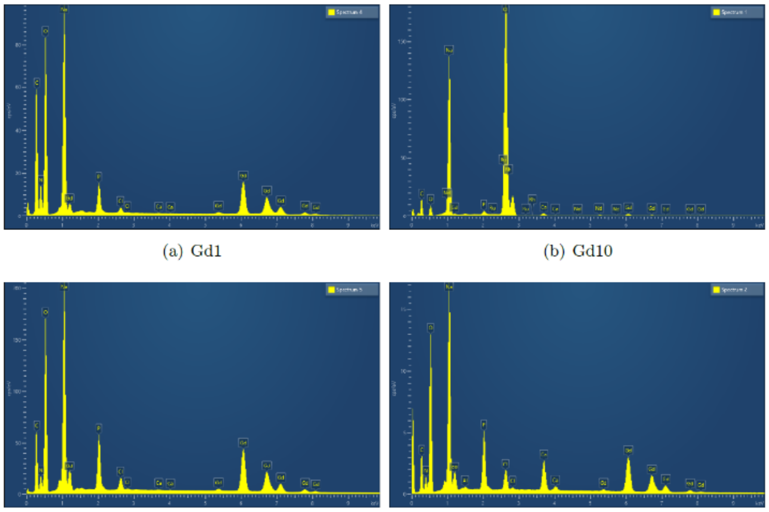

(a) Gd1 (b) Gd10

(c) Gd1 (d) Gd10

Figure S6. EDX results for HAp:Gd1 (a and c) and for HAp:Gd10 (b and d).

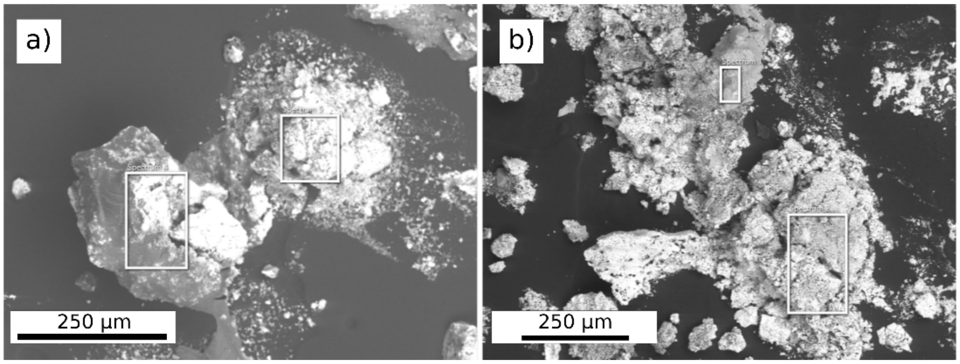

Figure S7. Scanning Electron Microscopy images of the two samples containing Gd. a) HAp:Gd1, b) HAp:Gd10. Inner rectangles represent the areas were the EDX technique was performed.
